# Three Microbial Musketeers of the Seas: *Shewanella baltica*, *Aliivibrio fischeri* and *Vibrio harveyi*, and Their Adaptation to Different Salinity Probed by a Proteomic Approach

**DOI:** 10.3390/ijms23020619

**Published:** 2022-01-06

**Authors:** Anna Kloska, Grzegorz M. Cech, Dariusz Nowicki, Monika Maciąg-Dorszyńska, Aleksandra E. Bogucka, Stephanie Markert, Dörte Becher, Katarzyna Potrykus, Paulina Czaplewska, Agnieszka Szalewska-Pałasz

**Affiliations:** 1 Department of Medical Biology and Genetics, University of Gdańsk, Wita Stwosza 59, 80-308 Gdańsk, Poland; anna.kloska@ug.edu.pl; 2 Department of Bacterial Molecular Genetics, University of Gdańsk, Wita Stwosza 59, 80-308 Gdańsk, Poland; grzegorz.cech@ug.edu.pl (G.M.C.); dariusz.nowicki@ug.edu.pl (D.N.); katarzyna.potrykus@ug.edu.pl (K.P.); 3 Laboratory of Phage Therapy, Institute of Biochemistry and Biophysics, Polish Academy of Sciences, Kładki 24, 80-822 Gdansk, Poland; monika.maciag-dorszynska@ug.edu.pl; 4 Laboratory of Mass Spectrometry-Core Facility Laboratories, Intercollegiate Faculty of Biotechnology UG and MUG, University of Gdansk, Antoniego Abrahama 58, 80-307 Gdansk, Poland; lewand.ola@gmail.com (A.E.B.); paulina.czaplewska@ug.edu.pl (P.C.); 5 Pharmaceutical Biotechnology, Institute of Pharmacy, University of Greifswald, Felix-Hausdorff-Str. 3, 17489 Greifswald, Germany; stephanie.markert@uni-greifswald.de; 6 Institute of Marine Biotechnology, Walther-Rathenau-Str. 49, 17489 Greifswald, Germany; dbecher@uni-greifswald.de; 7 Department Microbial Proteomics, Institute of Microbiology, University of Greifswald, Felix-Hausdorff-Str. 8, 17489 Greifswald, Germany

**Keywords:** osmotic stress, proteomic analysis, marine bacteria, *Shewanella baltica*, *Vibrio harveyi*, *Aliivibrio fischeri*

## Abstract

Osmotic changes are common challenges for marine microorganisms. Bacteria have developed numerous ways of dealing with this stress, including reprogramming of global cellular processes. However, specific molecular adaptation mechanisms to osmotic stress have mainly been investigated in terrestrial model bacteria. In this work, we aimed to elucidate the basis of adjustment to prolonged salinity challenges at the proteome level in marine bacteria. The objects of our studies were three representatives of bacteria inhabiting various marine environments, *Shewanella baltica*, *Vibrio harveyi* and *Aliivibrio fischeri.* The proteomic studies were performed with bacteria cultivated in increased and decreased salinity, followed by proteolytic digestion of samples which were then subjected to liquid chromatography with tandem mass spectrometry analysis. We show that bacteria adjust at all levels of their biological processes, from DNA topology through gene expression regulation and proteasome assembly, to transport and cellular metabolism. The finding that many similar adaptation strategies were observed for both low- and high-salinity conditions is particularly striking. The results show that adaptation to salinity challenge involves the accumulation of DNA-binding proteins and increased polyamine uptake. We hypothesize that their function is to coat and protect the nucleoid to counteract adverse changes in DNA topology due to ionic shifts.

## 1. Introduction

Osmotic stress comprises a relatively common environmental challenge encountered by bacteria in their natural habitats. Microorganisms, as the most adaptable organisms on Earth, have developed mechanisms to cope with the changes in osmotic conditions in order to maintain homeostasis for cellular processes. An increase or a decrease in cellular osmolality in response to alterations in the extracellular osmotic pressure may lead to a rapid water flux across the cell membrane to compensate for these changes; these events result in the concentration or dilution of the cytoplasm and lead to subsequent effects on cellular structures, enzymatic activity and metabolic pathways [1,2,3,4,5]. Microbial response to osmotic stress involves active changes in the concentration of specific osmoregulatory solutes [6,7]. These so-called compatible solutes can be accumulated up to molar concentrations without disturbing cellular processes, such as the metabolic pathways or DNA replication [1]. The identified osmolytes belong to various chemical groups: sugars (sucrose, trehalose), polyols (glycerol), amino acids (proline, glutamate), amino acid derivatives (peptides, proline betaine, ectoine), and quaternary amines (glycine betaine, carnitine) [5,8]. Interestingly, glycine betaine is a common osmolyte for both bacteria and marine invertebrates [9]. In the case of bacteria, it can be synthesized in the cells from choline or transported from the environment by the ProP and ProU transport systems [5,9]. 

Osmoregulation has been studied in several model bacteria, including *Escherichia coli*, *Bacillus subtilis* or *Lactobacillus plantarum* [5,9,10]. The response strategies may differ between bacterial species, however many mechanisms are common in various phylogenetic groups [10]. An immediate response to hyperosmotic shock involves potassium ion uptake using the Trk pathway, which is accompanied by uptake and synthesis of counterions, such as glutamate, to maintain electroneutrality [11,12]. Although this was mostly studied in detail with the *E. coli* model, potassium accumulation is generally associated with an early cellular response to the increase in osmotic pressure [13,14]. This serves as a signal for other processes, including regulation of compatible solute distribution [15,16]. This also includes active transport and synthesis of osmolytes in response to the osmotic upshift using specific transporters [5]. The uptake of the osmolyte precursors, e.g., choline, is accompanied by enzymatic synthesis of compatible solutes, such as glycine betaine [17]. The release of compatible solutes occurs under hypoosmotic conditions via mechanosensitive channels [9,18]. Homologs of the proteins involved in the activity of these mechanosensitive channels have been found in various phylogenetically distant bacterial species, which suggests high conservation of this system [19]. Still, multiple systems mediate osmoregulation by osmolyte accumulation and release. Many of them are regulated at the level of transcription, e.g., by two-component regulatory systems, or the assembled transporter activity [7,20]. 

The marine environment comprises a specific habitat in terms of high variability in physicochemical factors, including osmolarity and changes in the salinity levels. In particular, brackish water habitats and estuaries are the most challenging for microorganisms, where they are exposed to dramatic fluctuations in osmotic conditions. Despite such difficult conditions, the marine environment constitutes the largest habitat on Earth in terms of the area and the number of species. At a molecular level, the free-living bacteria’s response to changes in salt concentrations in their environment has been studied in bacterial species from various habitats, including *Caulobacter crescentus* (found in fresh water habitats)*, Vibrio vulnificus* (coastal waters)*, V. parahaemolyticus* (warm coastal waters), and *Salinispora* (marine sediments) [21,22,23,24,25,26]. Here, we chose three bacteria from distinct marine habitats: *Shewanella baltica* (found in both fresh and marine waters), *V. harveyi* (a facultative marine fish and invertebrate pathogen) and *Aliivibrio fischeri* (present in the temperate and subtropical global marine environments, also living as a squid symbiont), as models in our study.

*S. baltica* is a representative of the *Shewanella* genus, which is abundant in both marine and fresh water. This species was first isolated from the Baltic Sea, and later its role in fish spoilage was described [27]. Interestingly, the metabolic activity of *S. baltica* is also present at low temperatures, as spoilage of ice-stored fish was observed [27,28]. Recently, we investigated the response of *S. baltica* to low temperature at the transcriptional level [29]. *S. baltica* characteristics include its metabolic versatility, with the ability to use alternative electron acceptors, especially under anoxic conditions [30]. Additionally, it plays an important role in Baltic Sea denitrification due to its ability to reduce nitrates at the redox interface [31]. In addition to the flexibility of its metabolism, *S. baltica* adapts to various environmental challenges and stressors, such as nutrient limitation, DNA damage, and heat shock, by inducing a global stress response (the so-called stringent response) manifested by the accumulation of the (p)ppGpp alarmone [32].

*V. harveyi*, a Vibrionaceae family member, is a well-recognized and facultative bacterial pathogen of marine fish and invertebrates [33]. It is facultatively anaerobic, halophilic, and able to employ both fermentative and respiratory metabolisms. This bacterium exhibits quite a wide temperature range suitable for growth, however, warm waters are its main habitat, especially those in Asia, southern Europe, and South America [34]. Notably, salt is required for the efficient growth of *V. harveyi* both in natural and laboratory conditions [35]. *V. harveyi* cells communicate by using a quorum-sensing system which constitutes a model to study bacterial communication. As a pathogenic bacterium, *V. harveyi* is genetically and metabolically suited to increase its chance for survival under changing environmental conditions, such as in the presence of antibiotics or environmental pollution, which requires activation of specific protective mechanisms. Identification and description of such mechanisms may help to develop new methods to predict, prevent and control *Vibrio* infections of marine organisms [36,37].

Another example of a marine, Gram-negative bacterium is *A. fischeri*, distributed globally in temperate and subtropical marine environments [38]. *A. fischeri* has been described as a specific symbiont found in the light-emitting organs of certain squids and fish. The best-understood of the symbiotic associations of *A. fischeri* are those with sepiolite squids. These symbioses involve monospecific populations of *A. fischeri* cultured extracellularly, but within epithelium-lined crypts, in a specialized host organ. For example, *A. fischeri* employs a variety of regulatory proteins to produce polysaccharides and control biofilm formation within *Euprymna scolopes* [39]. The complex relationships between the host and bacterial symbiont are based on a mutual exchange of nutrients (including glucose, cAMP, chitin, peptides and several iron sources) and even the circadian rhythm [40,41]. Nevertheless, these metabolically flexible bacteria can be found either free-living or associated with a eukaryotic host. Living in and outside of a host likely requires the use of different metabolic pathways for bacterial growth and survival. The light-emission ability of *A. fischeri* is associated with the production of Lux proteins and is controlled via QS. Thus, this species constitutes a good model to study bacterial interaction and adjustment to changing environmental conditions.

In this study, we asked the question of how the three representative marine bacteria described above adapt to salinity changes, a common variation in their environment, to improve our understanding of the mechanisms ensuring their survival under various osmotic conditions. We aimed to reveal these bacterial global adaptation strategies at the proteomic level. We found that several biological processes were affected, including global gene expression regulation, transport, and central energy metabolism. We discuss and compare the response of the three bacterial species to osmotic stress and the role of specific processes in this adaptation.

## 2. Results and Discussion

### 2.1. Quantitative Analysis of Global Proteome Response to Salinity Challenge

**Figure 1:**
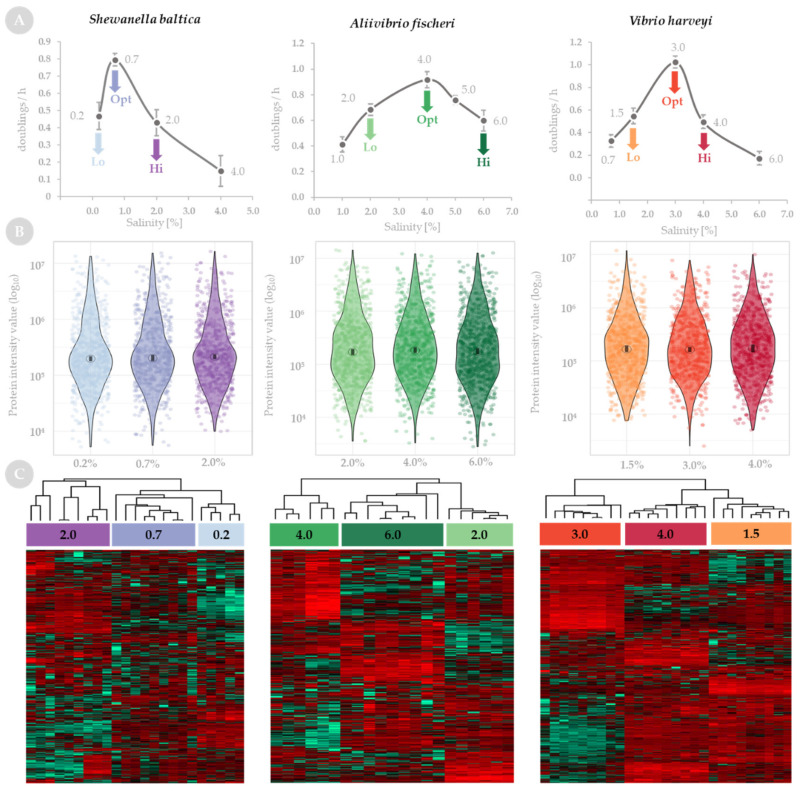
Bacterial growth and quality of protein samples identified by liquid chromatography with tandem mass spectrometry (LC-MS/MS) in *S. baltica*, *A. fischeri* and *V. harveyi* in response to salinity stress. (**A**) Growth rates determined for bacterial species cultured at different salt concentrations and presented as doublings per hour. Numbers on the plot correspond to salt concentrations at which these were assessed. Lo—low salinity, Hi—high salinity, Opt—optimal salinity. (**B**) Violin plots present the distribution of the abundance of all proteins detected by LC-MS/MS at low, optimal and high salinity. The percentage of salinity is presented on the x-axis. Obtained protein intensity values, normalized by a total area sums (TAS) approach are plotted on a log_10_ scale on the y-axis. Graphs show the data for each protein as jittered dots; an open circle indicates the median of the data and a vertical bar indicates for each median the 95% confidence interval determined by bootstrapping. (**C**) Heatmaps present protein expression at low, optimal and high salinity (percentage of salinity is marked above heatmaps); data are presented as z-score transformed, scaled in rows, values. Both biological and technical replicates are shown, and hierarchical clustering of replicates is presented above each heatmap.

We investigated the effect of challenges caused by nonoptimal salinity on proteomes of *S. baltica*, *A. fischeri* and *V. harveyi* by growing the bacteria at low, optimal and high salt concentrations. First, the bacteria were cultured at different concentrations of salt for 14–18 h and the optical density of the cultures was measured at 10 min intervals to assess the salinity requirements. Based on the calculated growth rates (Figure 1A), low, optimal and high salinity were determined as salt concentrations of 0.2, 0.7 and 2.0% for *S. baltica*, 2.0, 4.0 and 6.0% for *A. fischeri,* and 1.5, 3.0 and 4.0% for *V. harveyi*, respectively. We selected the low- and high-salinity points as the minimum and maximum salt concentrations at which bacteria could still grow and had a similar growth rate.

Protein samples extracted from bacterial cultures grown at low, optimal and high salinity were subjected to liquid chromatography with tandem mass spectrometry (LC-MS/MS) analysis. Three biological and three technical replicates were analyzed for each condition and were assessed for reproducibility with Pearson correlation; only replicates with strong correlation (Pearson’s r > 0.9) were included in the downstream analysis (Figure 1C). The principal component analysis (PCA) confirmed the clustering of the replicates (Supplementary Figure S1).

A total number of 684, 837 and 644 proteins were identified by LC-MS/MS for *S. baltica*, *A. fischeri* and *V. harveyi*, respectively (raw data are available in Supplementary Table S1). For quality control, we compared the distribution of the detected levels of all proteins (violin plots in Figure 1B). The distribution was comparable between the tested conditions for each species, confirming that the global expression levels detected in our experiments are normally distributed (not biased) and the samples we chose to further analyze for differential expression are normalized appropriately. Heatmaps in Figure 1C show the abundance of identified proteins and clustering between sample replicates for low, optimal and high salinity for each species. Interestingly, in *S. baltica*, proteomes from low and optimal salinity cluster, while in *A. fischeri* and *V. harveyi*, proteomes from the high and low salt concentrations are clustered (see heatmaps on Figure 1C).

**Figure 2:**
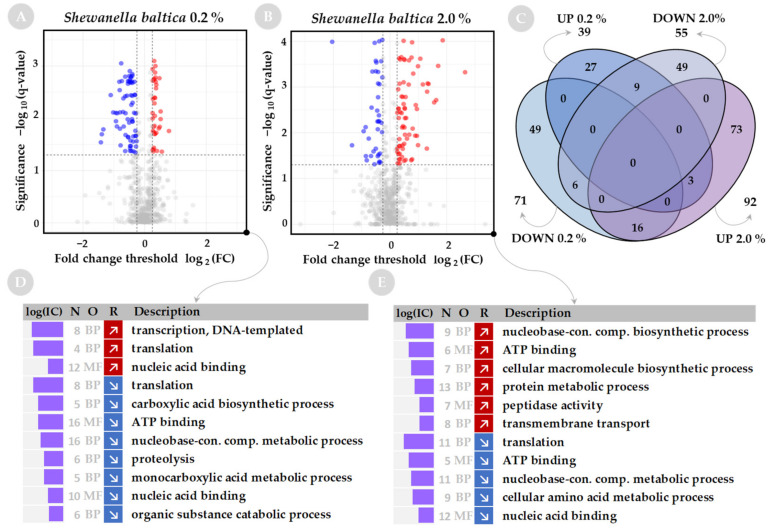
Global proteome changes in *S. baltica* in response to low (0.2%) and high (2.0%) salinity stress. Volcano plots presenting significantly up- and down-regulated proteins upon (**A**) low and (**B**) high salinity. Protein abundance, presented as log_2_-transformed fold change (FC) calculated versus protein abundance at optimal salinity, is plotted against the statistical significance presented as −log_10_-transformed *q*-value. Vertical and horizontal dashed lines indicate fold change thresholds set at |log_2_(FC)| ≥ 0.25 and −log_10_ (*q*-value) of < 1.3, respectively. Proteins with significantly increased and decreased levels are shown as red and blue dots, respectively. (**C**) Venn diagram showing numbers of significantly up- and down-regulated proteins at respective salinities; the total number of proteins with changed abundance is indicated outside each set. Annotation analysis of proteins with changed abundance upon (**D**) low and (**E**) high salinity. Schemes include the following columns: log-transformed information content (IC) score; the number of genes covered within the category (N); type of GO ontology (O)—biological process (BP) and molecular function (MF); type of regulation (R) with upwards pointing arrow on red background denoting upregulation, while downwards pointing arrow on blue background denotes downregulation of protein level; description denotes the name of GO terms retained in the analysis. The abbreviation nucleobase-con. comp. stands for the nucleobase-containing compound.

The identified proteins were filtered out with the fold change (FC) of at least 20% (|log_2_(FC)| ≥ 0.25) and adjusted *p*-value (i.e., *q*-value) < 0.05 was set as the minimum threshold for differential expression compared to samples obtained from bacterial cultures grown at optimal salinity. With these criteria, we observed that for *S. baltica*, low salinity (0.2%) results most prominently in downregulation of protein abundance (71 downregulated proteins vs. 39 upregulated), while at high salinity (2%) more of the differentially represented proteins are upregulated (55 downregulated proteins vs. 92 upregulated) (compare Volcano plots in Figure 2A,B). Most proteins with significantly changed levels are uniquely regulated at low or high salinity (Venn diagram on Figure 2C).

**Figure 3:**
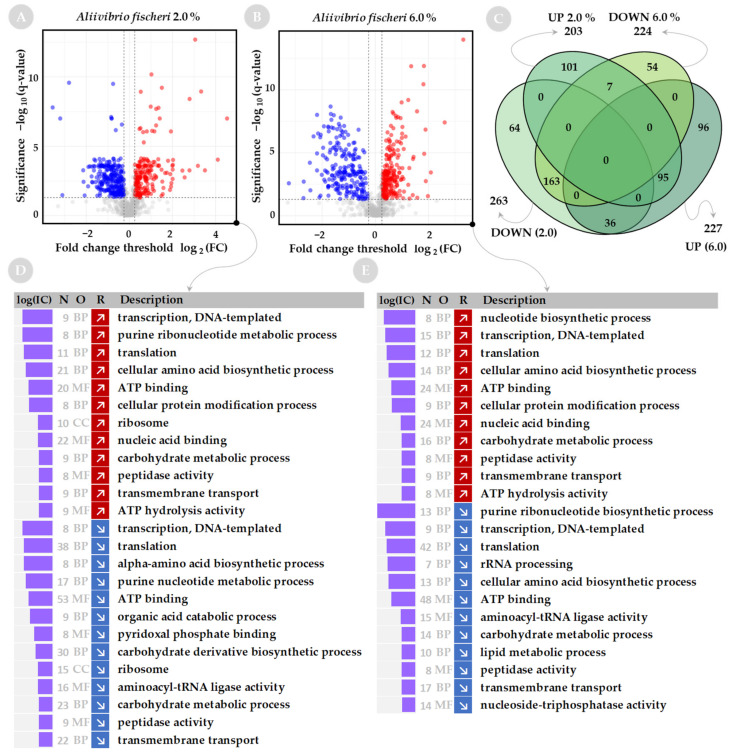
Global proteome changes in *A. fischeri* in response to low (2.0%) and high (6.0%) salinity stress. Volcano plots presenting significantly up- and down-regulated proteins upon (**A**) low and (**B**) high salinity. Protein abundance, presented as log_2_-transformed fold change (FC) calculated versus protein abundance at optimal salinity, is plotted against the statistical significance presented as −log_10_-transformed *q*-value. Vertical and horizontal dashed lines indicate fold change thresholds set at |log_2_(FC)| ≥ 0.25 and −log_10_ (*q*-value) of < 1.3, respectively. Proteins with significantly increased and decreased levels are shown as red and blue dots, respectively. (**C**) Venn diagram showing numbers of significantly up- and down-regulated proteins at respective salinities; the total number of proteins with changed abundance is indicated outside each set. Annotation analysis of proteins with changed abundance upon (**D**) low and (**E**) high salinity. Schemes include the following columns: log-transformed information content (IC) sore; the number of genes covered within the category (N); type of GO ontology (O)—biological process (BP), molecular function (MF), and cellular component (CC); type of regulation (R) with upwards pointing arrow on red background denoting upregulation while downwards pointing arrow on blue background denotes downregulation of protein level; description denotes the name of GO terms retained in the analysis.

**Figure 4:**
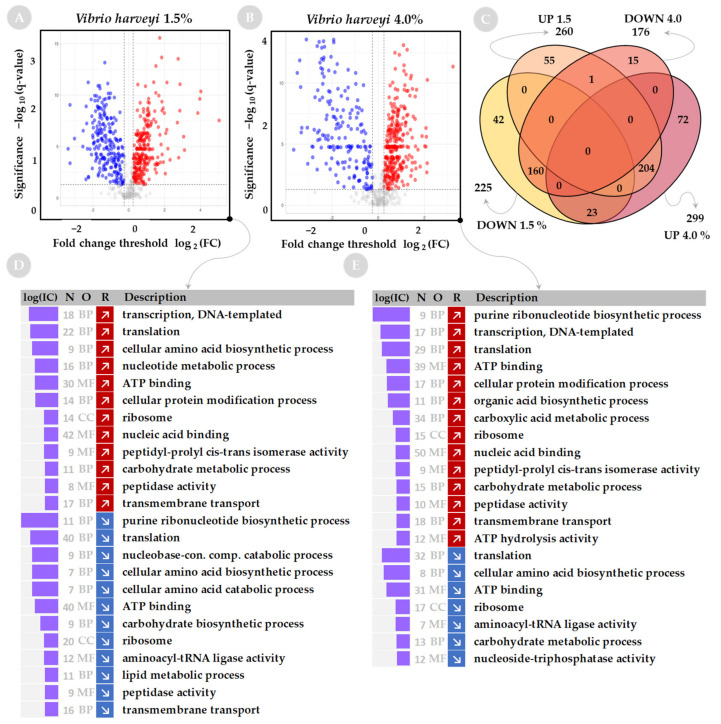
Global proteome changes in *V. harveyi* in response to low (1.5%) and high (4.0%) salinity stress. Volcano plots presenting significantly up- and down-regulated proteins upon (**A**) low and (**B**) high salinity. Protein abundance, presented as log_2_-transformed fold change (FC) calculated versus protein abundance at optimal salinity, is plotted against the statistical significance presented as −log_10_-transformed *q*-value. Vertical and horizontal dashed lines indicate fold change thresholds set at |log_2_(FC)| ≥ 0.25 and −log_10_ (*q*-value) of < 1.3, respectively. Proteins with significantly increased and decreased levels are shown as red and blue dots, respectively. (**C**) Venn diagram showing numbers of significantly up- and down-regulated proteins at respective salinities; the total number of proteins with changed abundance is indicated outside each set. Annotation analysis of proteins with changed abundance upon (**D**) low and (**E**) high salinity. Schemes include the following columns: log-transformed information content (IC) sore; the number of genes covered within the category (N); type of GO ontology (O)—biological process (BP) and molecular function (MF); type of regulation (R) with upwards pointing arrow on red background denoting upregulation while downwards pointing arrow on blue background denotes downregulation of protein level; description denotes the name of GO terms retained in the analysis. The abbreviation nucleobase-con. comp. stands for the nucleobase-containing compound.

Both *A. fischeri* and *V. harveyi* have a greater number of proteins with significantly increased or decreased levels at low or high salinity than was determined for *S. baltica* (Volcano plots on Figure 2A,B for *S. baltica*, Figure 3A,B for *A. fischeri*, and Figure 4A,B for *V. harveyi*). For *A. fischeri*, 463 and 451 proteins were affected at low and high salinity, respectively, and for *V. harveyi*, 485 and 475 proteins were affected, respectively, while for *S. baltica* only 110 and 147 proteins were affected at low and high salinities. In both Vibrios, a large pool of downregulated proteins (163 for *A. fischeri* and 160 for *V. harveyi*) or upregulated proteins (95 for *A. fischeri* and 204 for *V. harveyi*) is shared between corresponding salt conditions (see Venn diagrams on Figure 3C and Figure 4C).

Since shifts in salinity can be viewed as a global chemical abiotic stressor, the wider the salinity range, the more shifts in cellular response might be expected to be found, and it can be speculated that a wider salinity range requires more cellular adaptive responses. Indeed, this seems to be the case here, as the salinity concentration range allowing growth of the three bacterial species tested varies considerably, and there is a difference in the number of proteins and processes affected. The *S. baltica* range of salinity, with the least number of processes affected, is much narrower (0.2% to 2.0%) than that of *A. fischeri* (2.0% to 6.0%) and *V. harveyi* (1.5% to 4%), which display much more dramatic responses.

### 2.2. Functional Analysis of Global Proteome in Response to Salinity Challenge

Sets of up- and down-regulated proteins were subjected to annotation analysis. We used a web-based tool—GSAn—that combines analysis of GO terms by biological process (BP), molecular function (MF) and cellular component (CC) in a single run, and includes analysis of the information content of GO terms to explain how informative they are. The results of the GO term analysis are presented in Figure 2D,E for *S. baltica*, Figure 3D,E for *A. fischeri*, and Figure 4D,E for *V. harveyi*. A limited number of GO terms was found for *S. baltica* as the protein sets contained a smaller number of differentially represented proteins when compared with the two other species.

**Figure 5:**
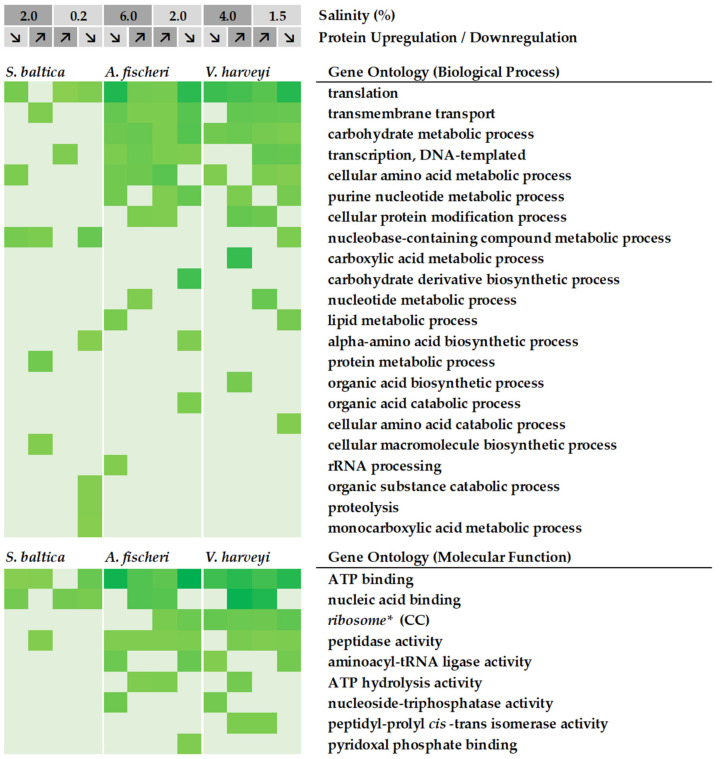
Summary of gene ontology analysis of proteins with differential abundance in *S. baltica*, *A. fischeri* and *V. harveyi* in response to salinity stress. Gray boxes indicate higher (Hi)- and lower (Lo)-than-optimal salinity conditions; the upwards- and downwards-pointing arrows indicate up- and down-regulation of proteins in each bacterium and condition. The hue of green color indicates the number of proteins that were obtained in annotation analysis shown in Figure 2, Figure 3 and Figure 4. The upper part of the heatmap shows biological process ontologies, and the lower part shows molecular function ontologies; * indicates an exception—the ribosome which belongs to the cellular component (CC) ontology. The higher on the list the gene ontology term is listed, the more abundant that category is in proteins.

We used those lists to generate a heatmap, shown in Figure 5, which summarizes the biological processes and molecular functions altered across the three bacterial species upon low and high salinity. In general, for all three bacteria, in the biological process ontology, differentially represented proteins most frequently annotate to GO terms such as translation (GO:0006412), transmembrane transport (GO:0055085), carbohydrate metabolic process (GO:0005975), transcription, DNA-templated (GO:0006351) or cellular amino acid metabolic process (GO:0008652). In the molecular function ontology, the most enriched terms include ATP-binding (GO:0005524), nucleic acid binding (GO:0003676), peptidase activity (GO:0008233), and aminoacyl-tRNA ligase activity (GO:0004812); while in the cellular component ontology, the term ribosome (GO:0005840) is the most enriched one. Similar GO terms exist for both low- and high-salinity conditions.

Bacterial cells adapt to changing environmental conditions by upregulation or downregulation of certain biological processes. The greater their adaptability in response to nonoptimal conditions, the greater the fitness of the organism. We have observed that bacteria adjust many biological processes in response to the salinity challenge, but *A. fischeri* and *V. harveyi* modulate a greater number of biological processes than *S. baltica*, thus, it can be speculated that their adaptability to changing salinity conditions is greater when compared with that of *S. baltica* and corresponds to greater fitness. A detailed discussion of the proteins assigned to the specified GO terms will be provided in the following paragraphs.

### 2.3. Changes in Salinity Affect the Nucleic Acid-Binding Proteins

We observed that for all three species of bacteria, the nucleic acid binding category is one of the most protein-abundant among molecular function GO terms (Figure 5). In general, we found that proteins annotated to this category are involved in biological processes such as transcription, RNA metabolism, replication, and DNA topology (even if some of these processes are not listed in Figure 5).

**Figure 6:**
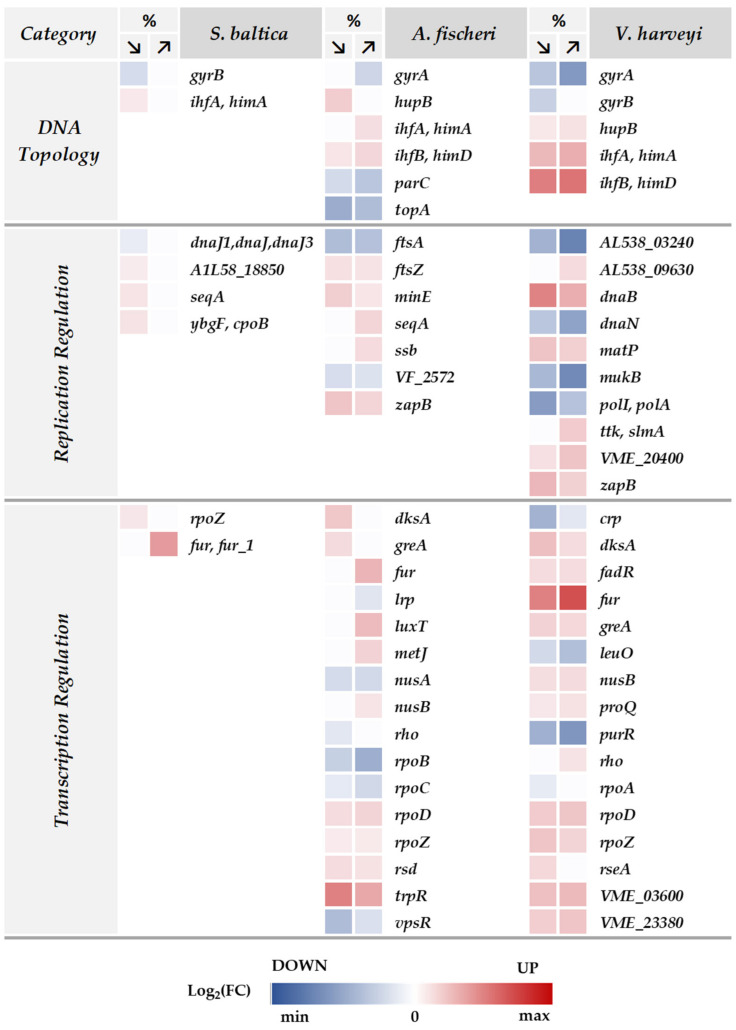
Heatmaps of the abundance of selected nucleic acid-binding, replication- and transcription-related proteins in *S. baltica*, *A. fischeri*, and *V. harveyi*, following low- and high-salinity stress. In the header, upwards- and downwards-pointing arrows indicate low- and high-salinity conditions, respectively. The hue of blue or red colors indicates the change in level (log_2_-transformed fold change (FC) values) of selected proteins relative to optimal salinity; the corresponding gene names are used to designate proteins. Exact protein fold change values can be found in Supplementary Table S2.

We found that in each strain, IhfA or IhfB subunits of the integration host factor (IHF), a chromatin regulator, are upregulated by either low salinity in *S. baltica* or low or high salinities in *A. fischeri* and *V. harveyi* (Figure 6, DNA topology category). IHF is a specific DNA-binding protein involved in genetic recombination, as well as in transcriptional and translational control. Interestingly, in Vibrios, IHF participates in transcriptional activation of quorum-sensing regulators [42]. In *V. harveyi*, IHF and LuxR coactivate quorum-sensing genes but also are required for transcriptional activation of the osmotic stress-response genes [43]. Indeed, in our study, we also observed upregulation of several proteins belonging to the Lux family (Supplementary Table S2). In *A. fischeri*, we identified upregulation of transcriptional regulators LuxT and LuxS, which are involved in the synthesis of the autoinducer used to communicate cell density and the environmental metabolic potential [43,44]. In *V. harveyi*, even more Lux proteins (LuxA, LuxB, LuxD, LuxE, LuxG, LuxH, LuxS) were upregulated. Therefore, one might speculate that there is a link between osmotic challenge and bacterial communication.

We also found increased levels of the HU protein, encoded by the *hupB* gene, upon salinity challenge in *A. fischeri* and *V. harveyi* (Figure 6). This protein is important for DNA compaction, replication, transcription, recombination, nucleoid structure maintenance, and shape modulation in many bacteria [45,46,47,48]. Interestingly, although HU binds all nucleic acids and their various hybrids in a sequence-unspecific manner [49], it exhibits a high affinity for abnormal DNA structures, generated, for example, in damaged DNA [50]. We also found that levels of the Hfq protein increase at low salinity in *S. baltica* and *V. harveyi* (log_2_(FC) = 0.29 and 0.50, respectively)*,* and at both salinities in *A. fischeri* (log_2_(FC) = 0.27 and 0.44 in low and high salinity, respectively) (Supplementary Table S2). Hfq is a well-known RNA chaperone that binds small regulatory RNAs (sRNAs) and mRNAs to facilitate mRNA translational regulation in response to envelope stress, environmental stress and changes in metabolite concentrations. Hfq also binds to DNA, alters its conformation, is considered as a nucleoid-associated protein (NAP) and can produce higher-organized structures [51,52,53,54]. Here, we assume that both HU and Hfq proteins may be involved in nucleoid organization and DNA protection in response to nonoptimal salinity challenge. DNA topology plays multiple roles in bacteria. It has been shown that high salinity has a stabilizing effect on nucleic acids, protecting biological macromolecules against heat degradation [55]. However, changes in environmental salinity have serious implications for DNA conformation. A reduction in hydration of the DNA molecule decreases the stability of its structure that is normally conferred by weakly bound water molecules [56]. For this reason, DNA-associating proteins are needed to preserve the proper structure of the bacterial nucleoid, so that gene expression is maintained and the cell can sustain its vital functions and growth.

On the other hand, we detected the downregulation of several topoisomerases. The abundance of gyrase subunits is lower in each tested bacterium upon nonoptimal salinity challenge (Figure 6). Interestingly, GyrB subunit is downregulated at low salinity in *S. baltica* and *V. harveyi*, while GyrA subunit is also downregulated at higher salinity in *A. fischeri* and *V. harveyi*. Additionally, we noted that two other topoisomerases—TopA and ParC—are downregulated in *A. fischeri* at both salinities tested. Gyrase imposes negative supercoils in closed circular dsDNA, maintains chromosomes in an underwound state, and catalyzes the interconversion of other topological isomers of dsDNA rings, including catenates and knotted rings. This activity is important, since negative supercoiling favors strand separation, DNA replication, transcription, recombination and repair [57]. TopA releases supercoiling and torsional tension of DNA, which are introduced during DNA replication and transcription, while ParC (subunit of topoisomerase IV) is essential for chromosome segregation, relaxes supercoiled DNA and performs decatenation events required during replication of a circular DNA molecule [58].

We also detected changes in the expression of several proteins directly involved in DNA replication (Figure 6). For example, we found upregulation of SeqA at low salinity in *S. baltica* and high salinity in *A. fischeri*. This protein is a negative regulator of replication initiation and ensures that this event occurs exactly once per chromosome per cell cycle. This implies that in *S. baltica* and *A. fischeri*, changes in salinity might lead to tighter regulation of replication initiation. While SeqA levels do not change in *V. harveyi*, several important replication proteins are less abundant in this bacterium upon salinity challenge, indicating decreased DNA replication as well. These include PolA (5′-3′ DNA polymerase with 5′-3′ and 3′-5′ exonuclease activity), DnaN (confers DNA tethering and processivity to DNA polymerases), and MukB (plays a central role in chromosome condensation, segregation and cell-cycle progression). On the contrary, levels of the DnaB helicase are increased in *V. harveyi* under both conditions, which might be surprising given the downregulation of the replicative proteins. However, it has been reported that certain types of ssDNA induce DnaB oligomerization into large complexes [59]; DnaB can also compact DNA similarly to the HU protein [60]. Thus, we speculate that DnaB may also be involved in maintaining the nucleoid architecture in response to the salinity challenge. Only in *S. baltica* in low salinity did we find downregulation of DnaJ, which actively participates in the response to hyperosmotic and heat shock conditions by preventing the aggregation of stress-denatured proteins and by disaggregating proteins. However, DnaJ is also involved in DNA replication through the activation of initiation proteins.

Additionally, the level of several cell-division proteins is affected in *A. fischeri* upon both salt conditions. For example, we detected increased levels of FtsZ (an essential cell division protein that forms a contractile ring structure—the Z-ring—at the future cell division site and recruits other cell-division proteins to the septum), MinE (prevents cell division inhibition), and ZapB (nonessential but abundant cell-division factor, required for proper Z-ring formation). On the other hand, levels of FtsA (responsible for the Z-ring assembly and anchoring in the membrane) were decreased. In the case of *V. harveyi*, ZapB levels were also increased under both salt conditions; also, the abundance of SlmA, a protein required for nucleoid occlusion and prevention of Z-ring formation through the nucleoid [61], was increased at high salinity.

To summarize, it seems that in response to osmotic stress, specific proteins gather around the nucleoid. This includes proteins involved in the maintenance of nucleoid structure and some proteins of the replication process, though only those that show the DNA-binding properties. The altered abundance of other proteins directly involved in replication or replication regulators suggests that the replication itself is inhibited. Interestingly, although bacterial cells accumulate several proteins involved in cell division, the cell division itself is also inhibited (we observe a decrease in growth rate upon salinity challenge). We speculate that we observe a well-orchestrated mode of action, in which bacteria inhibit the replication and cell-division processes and instead emphasize the protection and maintenance of the structure and function of the nucleoid under suboptimal salinity conditions.

The response to salinity challenge in *A. fischeri* and *V. harveyi* is generally the same regardless of the salt concentration used in experiments; this suggests a rather universal response to different environmental ionic shifts. Interestingly, in *S. baltica*, all changes occur only at a low salt concentration; moreover, the high salt concentration for this bacterium corresponds to the low salt concentration for the other two species. We might speculate that this points to a different strategy of adaptation to salinity changes in *S. baltica*. However, everything is compared to optimal conditions, so initially, *S. baltica* may have different protein levels than the other two strains.

### 2.4. Reprogramming of the Transcription Machinery as an Adjustment to Osmotic Changes

As the first line of response to most stressors and changes in the environment, bacteria adjust their transcription capacity to ensure optimal gene expression, and as a consequence, survival. Bacteria have a complex transcription regulation machinery encompassing the main enzyme, RNA polymerase, responding to numerous regulators. As unicellular organisms, bacteria need to make all adjustments quickly and accurately in the dynamic response to environmental alterations, thus transcription is the step when the effective reaction to stress can occur. In our studies, we assessed protein levels as a result of varied expression of their corresponding genes, which can be affected by post-transcriptional processes. However, we also observed changes in the number of proteins directly involved in transcription.

In promoter recognition, sigma factors, the interchangeable RNA polymerase subunits, play a crucial role. Thus, in the reprogramming of the transcription process, changes in the availability and levels of relevant sigma factors are often observed. Indeed, we observed an increased amount of the *rpoD* gene product, corresponding to the housekeeping sigma D subunit, in *A. fischeri* and *V. harveyi*, under both high and low salinity (Figure 6; Supplementary Table S2). This could indicate that transcription of the housekeeping genes is favored under these conditions. However, levels of the anti-sigma D proteins, products of VME_03600 in *V. harveyi*, and the *rsd* gene in *A. fischeri*, are also increased, which suggests tight regulation of sigma D availability.

Levels of other sigma factors are also altered during osmotic challenge. In *V. harveyi*, sigma 54 is more abundant, as well as the anti-sigma 28 factor (VME_23380) (Figure 6; Supplementary Table S2). However, the analogous alternative sigma 54 in *A. fischeri* is downregulated under both salinity conditions.

Interestingly, core RNA polymerase proteins, products of the *rpoB* and *rpoC* genes, are underrepresented under osmotic stress in *A. fischeri*, and the alpha subunit (*rpoA* gene product) is downregulated in *V. harveyi*. In contrast, the *rpoZ* gene product, encoding the omega subunit of the RNA polymerase, is upregulated in all three bacterial species. Omega is the only nonessential subunit of RNA polymerase, yet it has an important regulatory function, including in stress responses [62], thus its overrepresentation may illustrate the cell’s ability to combat stress that relates to osmotic challenges.

Changes in the transcription apparatus are not the only ones that we observed here in the global gene expression regulatory circuits. Levels of various proteins associated with the RNA polymerase are also altered under osmotic challenge (Figure 6; Supplementary Table S2). Namely, GreA and DksA are upregulated at both salinity conditions in *A. fischeri* and *V. harveyi*. These proteins affect RNA polymerase functions, in both transcription initiation and elongation, and their multiple roles in cellular processes have been described [63,64,65]. Especially, a role in stress response is assigned to DksA, including its important function in the stringent response [66,67]. The global stress response, mediated by a specific nucleotide, (p)ppGpp, is one of the first mechanisms activated during nutrient deprivation and numerous stresses in bacteria, as reviewed in [68]. Its induction would be an expected consequence of the osmotic stress, however we have shown previously that increased salt concentrations result in only a slight accumulation of the (p)ppGpp alarmone in *S. baltica* and *V. harveyi* [32]. This may be explained by the prolonged osmotic alterations: the bacteria in both experiments were cultured at altered salinity for several generations, thus, while initial stress could induce (p)ppGpp, the bacteria could reach the adaptation phase by the time of sample acquisition. Nevertheless, increased DksA levels indicate an enhanced ability of the cells to adjust to various transcription demands.

It is known that for the response to osmotic stress at the transcription level, the RpoS form of RNA polymerase is required (as reviewed in [7]). The reprogramming of the gene expression pattern is regulated by an increased glutamate level, which accumulates upon entry into osmotic stress [69]. In other stresses or during the transition into stationary phase, RpoS-dependent transcription requires (p)ppGpp facilitated by DksA [70]. Here, as the first phase of response to osmotic stress is potassium glutamate accumulation, the role of (p)ppGpp in *rpoS* induction could be dispensable.

Remarkably, *S. baltica* showed almost no changes in the transcription-associated protein levels. In addition to the above-mentioned *rpoZ*, only one protein level, A0A165IWX7 which is identified as an anti-sigma factor, was increased (Figure 6; Supplementary Table S2). This suggests that *S. baltica* may not need direct transcriptional machinery alterations to adjust to the osmotic changes, or that these alterations occurred mostly upon entry into the different salinity conditions, and later the transcriptional machinery stabilized at the initial level.

Among proteins involved in general transcription and affected by osmotic stress, there are regulators whose functions can be directly or indirectly related to chromosome replication, such as CspD, MatP and MetJ (Figure 6; Supplementary Table S2). CspD (cold shock-like protein) is upregulated at high salinity in *V. harveyi* and *A. fischeri*, and one of its roles is inhibition of chromosomal replication at the initiation and elongation steps during stress [71]. MatP, overrepresented under both osmotic conditions in *V. harveyi*, is involved in preventing chromosome segregation by binding to the Ter macrodomain [72]. MetJ (upregulated in high-salt conditions in *A. fischeri*), a repressor of the Met regulon, inhibits enzymes necessary for S-adenosylmethionine synthesis, thus indirectly slowing down the process of methylation of newly synthesized DNA [73]. These observations indicate that during osmotic challenge, DNA replication is repressed to minimize energy consumption by the cell.

**Figure 7:**
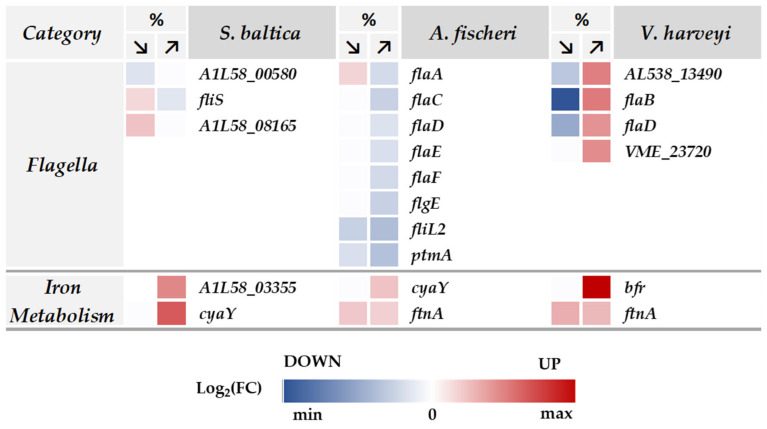
Heatmaps of expression of flagella- and iron metabolism-related proteins in *S. baltica*, *A. fischeri*, and *V. harveyi* following low- and high-salinity stress. In the header, upwards- and downwards-pointing arrows indicate low- and high-salinity conditions, respectively. The hue of blue or red colors indicates the change in level (log_2_-transformed fold change (FC) values) of selected proteins relative to optimal salinity; the corresponding gene names are used to designate proteins. Exact protein fold change values can be found in Supplementary Table S2.

Particular attention should also be paid, amongst other transcription regulators, to Fur. This protein plays a role in iron homeostasis, and in our study, we observed its upregulation in high-salt conditions in *S. baltica* and *A. fischeri*, and under both osmotic conditions in *V. harveyi* (Figure 6; Supplementary Table S2). Fur has a dual role and can act as a repressor of iron uptake and also as an activator [74]. Interestingly, high-salinity conditions were reported to cause iron limitation in *Bacillus subtilis* [75]. Indeed, we observed an upregulation of several proteins involved in iron uptake, namely CyaY, FtnA, Bfr (Figure 7; Supplementary Table S2). Notably, proteins involved in iron homeostasis were overrepresented in all three bacterial species tested.

Among the processes being regulated during the osmotic challenge is the ability of bacteria to move in the environment. This process requires the synthesis of specific proteins under global regulation. Proteins involved in bacterial motility, i.e., synthesis and regulation of flagella components, were also affected by the osmotic challenge (Figure 7; Supplementary Table S2). Interestingly, many flagellin proteins were downregulated under high-salt conditions in *A. fischeri*. At the same time, in *S. baltica* (low salt) and *V. harveyi* (high salt), upregulation of several flagellar components was observed. Bacterial motility was reported to be differently affected by osmotic stress. *Bacillus* swarming motility was impaired by high salinity [76] while *Salmonella enterica* flagellar gene expression was upregulated by hyperosmotic conditions [77]. The observed effect (multiple flagellar proteins affected by various osmotic conditions) suggests that motility and possibly chemotaxis play an important role in bacterial adaptation to salinity fluctuations.

Interestingly, flagellar motility is recognized as an important virulence factor in *V. harveyi* (reviewed in [37]). An increase in virulence of *V. harveyi* toward Indian prawn was evidenced at higher salinity levels [78]. Here, we show that in *V. harveyi* the levels of flagellin subunits that form filaments of flagella (AL538_13490, FlaB, and FlaD) and the flagellar protein FilL (VME_23720; a protein that controls the rotational direction of flagella) are increased only in high salinity (Figure 7; Log_2_(FC) is around 1.0). Moreover, we found that chitinase, another virulence-related factor that helps attach to the host and to penetrate its tissues [37], was upregulated in *V. harveyi* by high salinity (AL538_26130; Log_2_(FC) = 1.1) but was unchanged in low salinity (Supplementary Table S2). Other virulence factors, such as proteins involved in pilus assembly were downregulated in low salinity and unchanged in high salinity (AL538_07535 and SN10_09030; Supplementary Table S2). Thus, we hypothesize that high salinity might be primarily a trigger factor for this bacterium to increase its motility to find a potential host for colonization and prepare for adhesion. In natural environments, it could result in increased pathogenicity with increasing salinity.

### 2.5. The Effect of Osmotic Changes on Translation-Related Proteins

Translation is one of the downstream processes at which gene expression can be controlled. Thus, the ribosome density and occupancy status in the cell, together with mRNA level, actually control protein production. Our analysis shows that in bacteria grown under suboptimal salt conditions, one of the most prominently affected processes is translation (Figure 5). Changes in accumulation of enzymes involved in protein synthesis, in ribosomal proteins themselves and aminoacyl-tRNA (aa-tRNA) ligases, were observed (Supplementary Figure S2; Supplementary Table S2).

It is well-known that growing cells coordinate translation with metabolic rates, and the crucial element of this coordination is ribosome production. Ribosomes drive cell growth, but translation leading to the synthesis of ribosomal proteins competes with the production of nonribosomal proteins. We showed above that at suboptimal salinity, growth rates of strains employed in this study are significantly decreased (Figure 1A), implying the ribosomal cell content should also be decreased. Our proteomic analysis is in agreement with this assumption, revealing that the content of ribosomal proteins was generally downregulated at low and high salinity (Supplementary Figure S2; Supplementary Table S2). Intriguingly, Webber and Jung have also shown that hyperosmotic stress induces a significant decrease in the expression of genes encoding ribosomal proteins in *E. coli* [79]. However, in that study, this observation was correlated with high *rpoS* sigma factor expression and diminished expression of several genes involved in amino acid biosynthesis. As mentioned above, here we did not observe significant changes in RpoS accumulation nor in enzymes engaged in the amino acid synthesis pathway in low- and high-salt conditions. [80,81,82].

We found that, compared to the control cultures, in bacteria grown in low salinity, the numbers of downregulated proteins among those involved in translation regulation were one, five and seven, and upregulated were one, two and four for *S. baltica*, *A. fischeri*, *V. harveyi*, respectively. Under high-salt conditions, the numbers of downregulated proteins were one, six and seven for *S. baltica*, *A. fischeri*, and *V. harveyi*, respectively, and only five were upregulated for *V. harveyi*. We found that expression of the translation elongation factor (EF) protein family decreased at low and high salinity (Supplementary Figure S2; Supplementary Table S2). Namely, diminished expression of EF-Tu (*tufA*) in *S. baltica* and *A. fischeri*; EF g (*fusA*), EF-Ts (*tsf*), EF-4 (*lepA*) in *A. fischeri* and *V. harveyi* was observed. According to their role in aa-tRNA delivery to the ribosomes during peptide synthesis and GTPase activity, EF proteins are required to maintain proteome homeostasis in fast-dividing cells. It was also shown that the total rate of protein synthesis in bacterial cells growing exponentially is highly associated with both the translation elongation rate and the ribosome content [83,84].

Interestingly, following the reduction in EF levels, significant changes in abundance of aa-tRNA ligases were also observed (Supplementary Figure S2; Supplementary Table S2). Interestingly, in *S. baltica* and *A. fischeri*, underrepresentation of these enzymes was generally observed, while in *V. harveyi*, changes in both directions took place. The aa-tRNA ligases as carriers of amino acids in the process of protein synthesis are recognized by relevant EFs. Thus, we assume that their excess in cells during depressed translation would not be suitable for optimal energy metabolism under stress conditions. Nonetheless, the observed upregulation of specific enzymes could be linked with the synthesis of peptides utilized as osmolytes.

Taking all of the above into account, we show that the translation process and its machinery are generally affected under salinity challenge conditions in the three bacterial strains tested. Therefore, we assumed the adaptation of the protein synthesis process is a relevant bacterial mechanism to fit suboptimal salinity conditions.

### 2.6. Transmembrane Transport in Response to Salinity Stress

**Figure 8:**
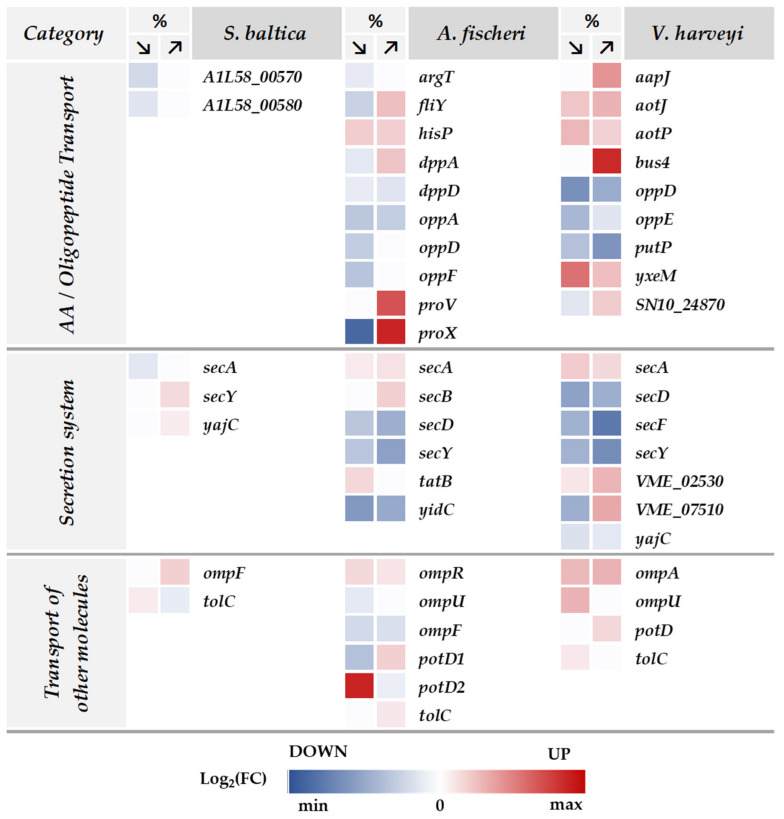
Heatmaps of expression of selected transmembrane transport proteins in *S. baltica*, *A. fischeri*, and *V. harveyi* following low- and high-salinity stress. In the header, upwards- and downwards-pointing arrows indicate low- and high-salinity conditions, respectively. The hue of blue or red colors indicates the change in level (log_2_-transformed fold change (FC) values) of selected proteins relative to optimal salinity; the corresponding gene names are used to designate proteins. Exact protein fold change values can be found in Supplementary Table S2.

As a general response to osmotic stress, bacteria adjust the content of their cells not only by synthesis/degradation balance, but importantly, by transporting compounds in and out through their membranes. Our proteome analysis revealed that in *A. fischeri* and *V. harveyi*, many differentially expressed proteins annotate to the GO transmembrane transport (GO:0055085) term (Figure 5). In response to osmotic stress, both species modulate the level of several transporter proteins of the ATP-binding cassette (ABC) transporter superfamily (including compatible solute, polyamine, or amino acid transporters), proteins of the bacterial secretion system, outer membrane proteins, and ion transporters (Figure 8; Supplementary Table S2). Such prominent changes were not observed for *S. baltica*.

Among the proteins with the most differential expression in response to the osmotic challenge, we found transporters of compatible solutes. Accumulation of these compounds in the cell results in the influx of water, and thus the bacterial cell ameliorates the effects of high external osmolality [85]. Compatible solutes also act as protectants to proteins and nucleic acids.

At high salinity, we observed a greatly increased accumulation of the glycine betaine transporter subunits ProX (VF_0785) and ProV (VF_0787) in *A. fischeri* and a protein annotated as a substrate-binding domain of ABC-type glycine betaine transport system family, encoded by the *bus4* gene (AL538_17540; amino acid sequence homology to ProX) in *V. harveyi* (Figure 8). Additionally, a protein annotated as containing an OpuAC domain (VME_07510), that has a function assigned to choline transport and choline binding, was upregulated at high salinity in *V. harveyi*. While glycine betaine is a well-known osmoprotectant, choline is a metabolic precursor for glycine betaine biosynthesis, but it was also recognized to have a strong osmoprotective effect itself (e.g., in *Pseudomonas syringae*) [26]. Intracellular accumulation of compatible solutes is obtained either by uptake from the environment or de novo biosynthesis, and transport of these solutes is much less energy-consuming in comparison to their biosynthesis [26,85]. Indeed, under our experimental conditions, we observed an increased abundance of compatible solute transporters in response to high salinity, indicating that osmolyte uptake takes precedence over biosynthesis, as a less energy-consuming response to changing osmolarity. However, at the same time in *A. fischeri*, the EctB protein, involved in the ectoine biosynthetic process is upregulated by high salinity (log^2^(FC) = 2.09; Supplementary Table S2). Thus, it seems that both routes are activated at the same time to achieve a rapid and efficient adaptation to high external osmolality.

In hypoosmotic environments, compatible solutes must be removed from the cell to counteract the influx of water and rapid turgor increase. Excretion of compatible solutes in response to low osmolarity is achieved by the opening of mechanosensitive channels, but other mechanisms, such as multidrug-resistance-type exporters or substrate-specific efflux systems are hypothesized [85]. We found that the expression of compatible solute transport proteins described above generally did not change in response to low osmolality when compared to optimal conditions. Accumulation of the periplasmic protein ProX that binds to glycine betaine/proline betaine is the only one that shows a strong reduction in *A. fischeri* (Figure 8), indicating that restriction of compatible solute uptake may be achieved simply by decreased substrate binding in the periplasmatic space.

In addition, an interesting process regulated by the osmotic stress that we identified in *A. fischeri* and *V. harveyi* is the transport of polyamines (GO:0015846). In *A. fischeri*, accumulation of the putrescine-binding periplasmic protein (VF_1318; PotD2), a component of the bacterial periplasmic transport system of putrescine and spermidine, is strongly upregulated at low salinity but downregulated at high salinity (Figure 8). Conversely, the level of PotD1 (VF_1319), also assigned as the putrescine-binding periplasmic protein (a key component of the polyamine uptake system—PotABCD), is upregulated at high salinity but downregulated at low salinity. Similarly, PotD is overrepresented in *V. harveyi* at high salinity. Polyamines play various roles in cells and are associated with the response to different types of stress (reviewed in [86,87,88]). Since they are positively charged at physiological pH, polyamines can function as reactive oxygen species (ROS) scavengers or bind to negatively charged molecules, providing stabilization and protection of nucleic acids or proteins. They act as osmolytes, regulate the Na^+^ influx and K^+^ efflux or bind to porins to inhibit their activity and counteract the negative effects of osmotic stress on the cell. Interestingly, exogenous putrescine may also be utilized by transamination pathways to succinate and thus supply the nitrogen for the synthesis of alanine and glutamate [89]. Accumulation of potassium glutamate is one of the first responses of bacteria to hyperosmotic stress, which serves as transient protection and also provides reprogramming of bacterial gene expression patterns [12,69].

Polyamines are derived from ornithine and methionine, but arginine and lysine serve as secondary sources for the production of these metabolites. We also observed changes in the expression of amino acid transporters in response to the osmotic challenge. Several amino acid ABC transporter proteins show differential abundance upon nonoptimal salinity (Figure 8; Supplementary Table S2). In *A. fischeri*, we observed upregulation of histidine/lysine/arginine/ornithine transporter subunit (HisP) under both tested conditions and cystine-binding protein (FliY) upregulation in high salinity and downregulation in low salinity. In *V. harveyi*, we identified an increased expression of two proteins belonging to the arginine and ornithine transport system, that is, the substrate-binding protein AotJ (VH1709_contig00084-0056) and the ATP-binding protein AotP (VME_17230) (Figure 8). Additionally, the general L-amino acid transport system substrate-binding protein (VME_48840, with amino acid homology to AapJ, BztA) and a putative S-methylcysteine transport system substrate-binding protein (AL538_09685, assigned to YxeM) are upregulated at low and high salinity. Interestingly, the PutP protein, which catalyzes the sodium-dependent uptake of extracellular L-proline, is highly downregulated at low and high salinity in *V. harveyi*. Among other compatible solutes, arginine is one of the osmoprotectants responsible for osmotic adjustment to tissue dehydration in plants, and ornithine is an intermediate in the arginine biosynthesis pathway [90]. Both can act as precursors of polyamines that also play a role in plant protection from osmotic stress. Perhaps the increased expression of transporters of these amino acids in bacteria may be also involved in adaptation to osmotic challenge.

Additionally, we observed major downregulation of components of the oligopeptide transporter system at both salinities (Figure 8), which suggests that the uptake of oligopeptides may be suppressed during osmotic shock. These include OppD (*A. fischeri* and *V. harveyi*); OppA, OppF, DppA, and DppD (*A. fischeri*), and OppE (*A. fischeri*). The Opp system in bacteria is involved in nutrient uptake, recycling of cell-wall peptides or sensing of signaling molecules.

Upon salinity challenge applied to all three bacterial species tested, we also found differences in the amounts of proteins participating in protein secretion systems. These are the SecA and signal recognition particle (Sec-SRP) and the twin-arginine targeting (Tat) translocons (Figure 8). In the Sec-SRP system, unfolded proteins are delivered from the cytoplasm to the translocon and subsequently folded in the periplasm. In contrast, the Tat system translocates only fully folded proteins across the periplasmic membrane [91]. We found that in *A. fischeri* and *V. harveyi*, SecD, SecF, SecY (anchored in the periplasmic membrane) and YajC (an insertase) are underrepresented at both salinities, while the cytoplasmic proteins SecA and SecB are moderately upregulated under these conditions. On the other hand, we observed that the TatB protein is slightly overrepresented at low salinity in *A. fischeri*. Our results indicate that the export of proteins through the periplasmic membrane is downregulated in response to salinity challenge. As the translation itself is also downregulated (discussed in chapter 2.5), it appears that the cellular secretion systems are also gradually shut down and the export of proteins through the cell membrane is inhibited. Interestingly, *S. baltica* responds to osmolality changes by the accumulation of Sec-translocon proteins (SecY and YajC) at high salinity and downregulation of SecA at low salinity. These differences might be related to either different genus-specific adaptation strategies or the fact that *S. baltica* was tested under a much narrower salinity range than the other two bacterial species.

In addition, upon osmotic stress conditions, we found that several Omp proteins show differential abundance, such as OmpF (increased in *S. baltica* (A1L58_00615); decreased in *A. fischeri* (VF_A1164)), and OmpA (increased in *V. harveyi* and *S. baltica* (A1L58_16290)) (Figure 8; Supplementary Table S2). These outer membrane proteins play an important role in membrane permeability to solutes and proteins and are crucial for signal transduction. Additionally, TolC, an outer membrane protein that plays an important role in the export of toxic substances, cell growth, biofilm formation and is known to contribute to tolerance to osmotic stress, was overrepresented in all three species in low- and high-salt conditions.

### 2.7. Chaperones as a Response to Osmolarity Alterations

**Figure 9:**
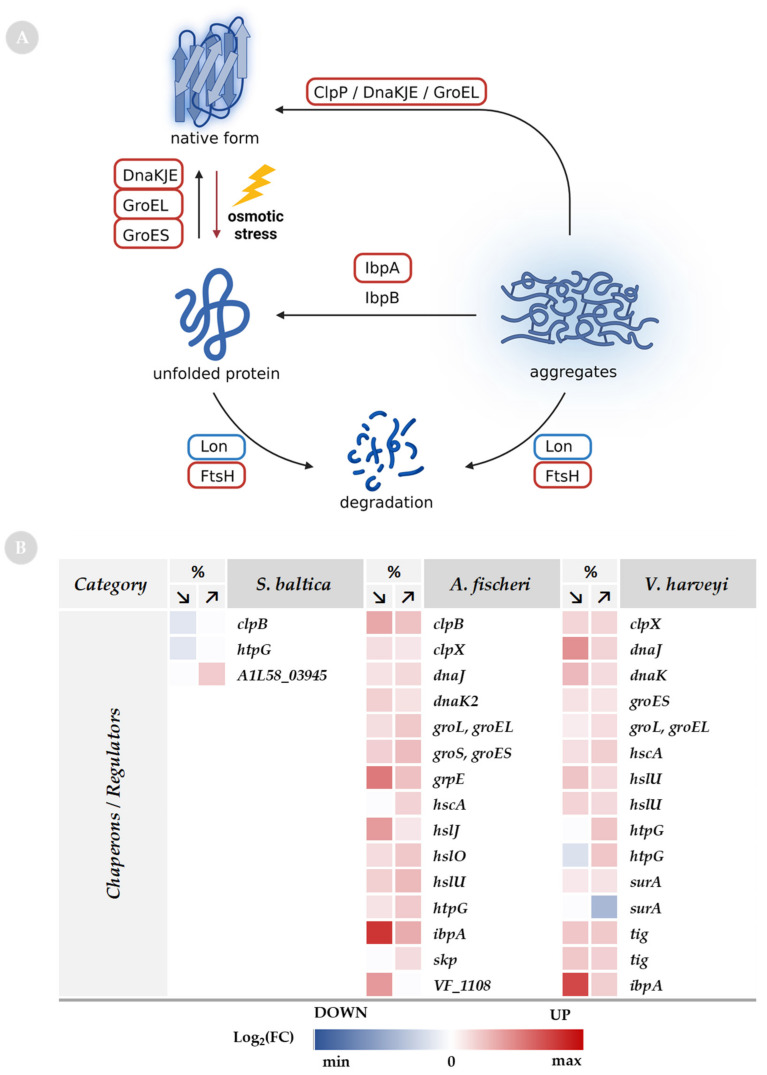
Osmotic stress induces response to misfolded and aggregated proteins in *A. fischeri* and *V. harveyi*. (**A**) Schematic representation of the recruitment of chaperones and proteases to maintain cellular proteostasis. The upregulated proteins are marked in red boxes and the downregulated ones in blue boxes. (**B**) Heatmaps of the abundance of chaperone proteins in *S. baltica*, *A. fischeri*, and *V. harveyi* following low- and high-salinity stress. In the header, upwards- and downwards-pointing arrows indicate low- and high-salinity conditions, respectively. The hue of blue or red colors indicates the change in level (log_2_-transformed fold change (FC) values) of selected proteins relative to optimal salinity; the corresponding gene names are used to designate proteins.

Osmotic stress is a great challenge in maintaining cellular proteostasis. The bacterial adaptation mechanism to suboptimal salinity is based on the regulation of the intracellular osmolytes level (described above) and activation of chaperone pathways that promote the folding of native proteins. In addition to preventing protein aggregation, chaperone proteins mediate aggregate clearance through proteolysis of non-native proteins and aggregation reversal [92]. Indeed, in this study, we found that proteins such as chaperones and proteases involved in protein maturation and degradation were differentially regulated due to osmotic challenge in *A. fischeri* and *V. harveyi* (Figure 9). Namely, the levels of DnaK/DnaJ/GrpE, GroEL, ClpB, and IbpA were increased under low- and high-salt conditions. These proteins are essential for the effective processing of aggregated proteins, by either refolding them to their native form or unfolding them for degradation [93]. Interestingly, while the Lon protease has been reported to be the most specific for the aggregate degradation process, our analysis shows that the Lon enzyme remains downregulated in *A. fischeri* and unchanged in *V. harveyi* upon suboptimal salinity. Thus, we conclude that under osmotic stress, unfolded proteins are preferentially refolded by chaperones rather than degraded by proteases. However, we also observed an elevated level of HslU unfoldase and FtsH protease, members of the AAA+ protease family, and other small peptidases such as amino- and carboxy-peptidases. The important roles in the protein quality control of HslU and FtsH enzymes are well-described [94,95]. FtsH, as well as GroEL/GroES and DnaK/DnaJ/GrpE, are also involved in maintaining the balance of sigma factors in cells [95,96]. However, growth under stress conditions requires optimization of the synthesis processes and maintenance of the availability of building components, which can be achieved by enhanced proteolytic activity. Thus, we assumed that, in general, chaperones are more abundant than proteases under stress conditions, hence refolding is favored over degradation (Figure 9). It is noteworthy that, to date, little is known about chaperone–protease complex involvement in proteostasis in low-salinity conditions.

### 2.8. Metabolic Adjustment to Salinity Changes

The global reprogramming of the bacterial cellular processes to the numerous stresses also affects cellular metabolism. As a consequence of other adjustments at various levels, the central carbon metabolism in particular could also be affected, as shown for *B. subtilis* [97]. This comprises a series of processes leading to the production of energy through the transformation of substrates and their intermediates, necessary for the development and reproduction of cells. Intermediate metabolites obtained from these transformations constitute a pool of starting substrates for the macromolecular synthesis pathways. Glycolysis, gluconeogenesis, the pentose phosphate pathway, and the tricarboxylic acid cycle form the core of the carbon metabolism pathways, while glucose is the most common substrate used in energy reactions that underpin the functioning of the entire cell. Nevertheless, depending on the environmental conditions, cells can use various organic compounds, incorporating them into pathways at relevant stages.

**Figure 10:**
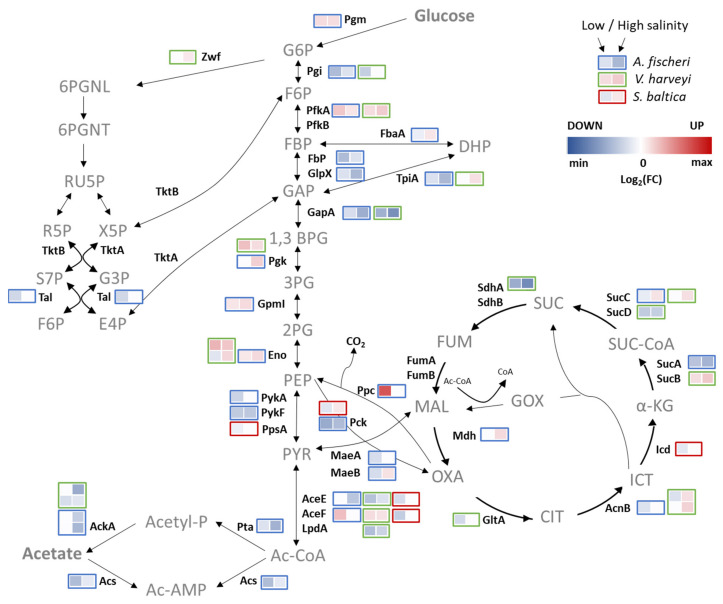
Metabolic adjustment to salinity changes. Central carbon metabolism scheme with heatmaps of abundance of selected cellular metabolism-related proteins in *S. baltica* (red frame), *A. fischeri* (blue frame), *V. harveyi* (green frame) following low- and high-salinity challenge (left and right site of a frame, respectively). The hue of blue or red colors indicates the expression level (log_2_-transformed fold change (FC) values) of selected proteins relative to optimal salinity. Exact protein fold change values can be found in the Supplementary Table S2. Metabolite abbreviations: 1,3-BGP, 1,3-biphosphoglycerate; 2PG, 2-phophoglycerate; 3PG, 3-phosphoglycerate; 6PGLN, 6-phosphoglucono-δ-lactone; 6PGNT, 6-phophogluconate; GLC, glucose; G6P, glucose-6-phosphate; F6P, fructose-6-phosphate; FUM, fumarate; MAL, malate; OXA, oxaloacetate PBP, fructose-1,6-biphosphate; DHAP, dihydroxyacetone phosphate; GAP, glyceraldehyde 3-phosphate; PEP, phosphoenolpyruvate; PYR, pyruvate; Ru5P, ribulose-5-phosphate; R5P, ribose-5-phosphate; S7P, sedoheptulose-7-phosphate; E4P, erythrose-4-phosphate; Ac-CoA, acetyl coenzyme A; Ac-P, acetyl phosphate; Ac-AMP, acetyl-AMP; CIT, citrate; ICT, isocitrate; GOX, glyoxylate; a-KG, α-ketoglutarate; SUC-CoA, succinyl-coenzyme A; SUC, succinate; Xu5P, xylulose-5-phosphate.

The global bacterial response to high-salinity-environment exposure leads to many changes in the cellular level of enzymes, metabolic intermediates, and other processes. Changes in the proteomic profile of *V. harveyi*, *A. fischeri*, and *S. baltica* responding to low salt concentrations or hyperosmotic stress present some essential information (Figure 10). Downregulation of CCM enzymes suggests that the carbohydrate metabolism of all tested bacteria is impaired when these organisms are exposed to osmotic stress or salt deficit. Because cellular adaptation to a high-salinity environment is an energy-consuming process, several upregulated proteins suggest that the cells try to restore energy from all metabolic components. Changes in the expression profiles of glycolytic enzymes under stress were already reported for other bacteria. For example, in *Lactobacillus lactis*, changes in glycolytic enzymes suggest that different energy demands depend on a variety of stresses. Upregulation of glycolytic proteins may also increase the NADH/NAD^+^ ratio [98].

We find here that in *S. baltica*, one protein was upregulated under both conditions, eight were downregulated in low salt conditions and two in high salt conditions. This suggests that the cells had slowed down their metabolism under adverse environmental conditions. All proteins whose levels were changed belong to the pyruvate synthesis and metabolism pathway. An induction was observed for the phosphoenolpyruvate carboxykinase (Ppc), an enzyme involved in gluconeogenesis during the energy and carbon sourcing of TCA components. Ppc catalyzes the conversion of oxaloacetate to phosphoenolpyruvate. At low salt concentrations, two components of the pyruvate dehydrogenase complex (AceE and AceF) were downregulated, along with the beta subunit of succinate-CoA ligase. At low salt concentration, we also observed downregulation of isocitrate dehydrogenase, catalyzing the oxidative NAD(P)(+)-dependent decarboxylation of isocitrate to alpha-ketoglutarate and CO_2_. In the two other bacteria, more proteins from the glycolysis pathway were changed. In *A. fischeri*, six enzymes that convert fructose-6-phosphate to glyceraldehyde-3-phosphate, involved in the beginning of the glycolysis process, were upregulated. Enzymes that convert glyceraldehyde-3 phosphate to pyruvate were also induced. On the other hand, phosphoenolpyruvate synthetase (PpsA), an enzyme that catalyzes the phosphorylation of pyruvate to phosphoenolpyruvate, was induced only at high salt concentrations, similarly to phosphoglycerate kinase (Pgk). Five glycolytic/gluconeogenic enzymes were downregulated in both environmental conditions. Additionally, fructose-bisphosphate aldolase (FbaA) and phosphoenolpyruvate carboxykinase (Ppc) were also downregulated, but only at low salt concentrations.

In the case of *V. harveyi*, four proteins were upregulated in both conditions, while four were increased only at high, and three at low salt concentrations. Five proteins, including two reduced at both conditions and two in low-salt conditions, were downregulated. Interestingly, it is known that in high salt environmental conditions, the carbon metabolism diverts to osmolyte synthesis [97,99]. One of the osmolytes is trehalose. Although in Vibrionaceae, biosynthesis of trehalose was not described, *Vibrio* cells can use trehalose as a carbon source [26]; upregulation of trehalose-6-phosphate hydrolase (TreC), an enzyme that converts trehalose-6-P to glucose-6-P and glucose, in *V. harveyi* in high-salt conditions confirms this hypothesis. Changes in the level of other glycolytic enzymes in *A. fischeri* and *V. harveyi* might be an effect of energy obtained from other metabolic intermediates, for example, intermediates from the TCA cycle. This pathway is used to produce precursors for the amino acids, purines, pyrimidines, vitamin biosynthesis, and NADH is supplied for the redox reactions. Observed changes in TCA-cycle protein levels, in all tested bacteria, suggest that this pathway is a key element in adaptation to changes in salinity level. The altered NADH/NAD ratio under salt stress was also reported for other bacteria, such as *E. coli*, *Lactobacillus lactis* and *Zymomonas mobilis* [98,100].

Pyruvate, as a product of a glycolytic process and initial substrate in the TCA cycle, is converted into acetyl-CoA and carbon dioxide by pyruvate dehydrogenase under aerobic conditions. This suggests that changes in the AceE and AceF levels (components of the pyruvate dehydrogenase complex) may have a role in adaptation to changing salinity, since in *A. fisheri*, AceE was downregulated in high salt and upregulated in low salt concentrations. On the other hand, AceE was downregulated and AceF was upregulated in *V. harveyi* at low and high salt concentrations. Additionally, in *V. harveyi*, the third component of the pyruvate dehydrogenase complex was affected—the dihydrolipoyl dehydrogenase (LpdA), a component of two types of dehydrogenases. Along with oxoglutarate dehydrogenase (SucA) and dihydrolipoyllysine-residue succinyltransferase (SucB), Lpd forms an alpha-ketoglutarate dehydrogenase complex, and with AceE and AceF it forms a pyruvate dehydrogenase complex. Lpd was downregulated in both conditions. Additionally, four proteins from the TCA cycle were upregulated at high salt concentrations and four downregulated at low salt concentrations.

In *A. fischeri*, the transketolase (TktB) and transaldolase (Tal) involved in the pentose phosphate pathway were reduced in low-salt conditions, while transketolase was also reduced in high-salt conditions. In the case of *V. harveyi*, glucose-6-phosphate 1-dehydrogenase was induced at high salt concentrations and transaldolase at low salt concentrations. No proteins from the pentose phosphate pathway were altered in *S. baltica*, and no effect on the protein level was observed in the acetate metabolism. Still, the acetate kinase, as a component of the acetate metabolism, was downregulated at high and at low salt concentrations.

In *A. fischeri*, the level of acetate kinase (AckA) was reduced only at a high salt concentration. Interestingly, additional proteins from the acetate metabolism were downregulated in this bacterium under both salt conditions, namely the phosphate acetyltransferase and acetyl-CoA synthase. In *E. coli*, the main pathway leading to acetate production involves two CCM enzymes: phosphotransacetylase (Pta) and acetate kinase (AckA). Acetyl-CoA, the product of glycolysis and the consumable substrate for the tricarboxylic acid (TCA) cycle, can be converted into acetyl-phosphate (AcP) by Pta and then into acetate by AckA in the exponential phase of bacterial growth [101]. AcP, as a high-energy molecule, regulates many cellular processes in Enterobacteria by phosphorylating or acetylating proteins and other molecules. Pta and AckA modulate intracellular levels of this molecule [101].

### 2.9. The “All-Hands-on-Board” to Protect the Nucleoid Hypothesis

Each aquatic environment is a complex ionic solution. In the sea, ionic composition changes upon rapidly occurring conditions, such as storms, weather breaks, floods, low-water exchange, etc. These environmental events may create shifts in composition and ion strength of the local solution environment. Each bacterial cell is also a complex ionic solution containing different macromolecules, including proteins and DNA. In bacterial cells, the nucleoid is a complex architecture and can be instantly adapted for specific purposes [102] and the ions and the ionic strength of a solution have a great influence on the DNA topology. Hypothetically, in an evolutionary context, this could be considered as an arms race between the *ionic conditions* and the *DNA-focused interests*. Additionally, the DNA’s primary “*interests*” are always stability and protection. In our study, we observed that upon nonoptimal salinity conditions, cell adaptation involves the accumulation of DNA-binding proteins, and many of these proteins increase negative DNA supercoiling. Additionally, the increased abundance of polyamine transporters suggests an increased polyamine uptake in response to the osmotic challenge. Among other functions, polyamines interact with nucleic acids, stabilize and protect DNA conformation, participate in packaging and contribute to gene expression regulation [86,103]. It seems that the DNA needs to be covered and secured to be functional; otherwise, every ionic “*storm*” (too high or too low salinity) might disrupt some DNA-related processes or even cause physical damage. Indeed, among differentially expressed proteins, we found many DNA-binding proteins which are components of the replication machinery. This should not be surprising if we consider that many of these proteins have more than one function. On the one hand, we can interpret this phenomenon as “*all-hands-on-board*” *to protect the nucleoid*—any DNA-binding protein will be better than none at the brink of undesirable and unsupervised DNA-topology changes. However, on the other hand, another scenario might be hypothesized—upregulation of proteins modulating the nucleoid architecture or proteins forming higher-organized structures can be viewed as a protective scaffold and factors maintaining the exact desired DNA topology. Certain DNA supercoiling promotes the expression of specific genes, and many of the DNA-binding transcriptional factors rely on local topology. Adding to this scenario the likely increased transport of polyamines and their protective role for DNA, we can speculate that the cell activates an efficient mode to maintain DNA’s stable structure and function upon nonoptimal salinity challenge.

## 3. Materials and Methods

### 3.1. Bacterial Strains and Growth Conditions

The following bacterial strains were employed in this study: *S. baltica* M1 [104], *V. harveyi* BB7 [105] and *A. fischeri* ES114 [38]. Bacteria were cultivated in liquid Instant Ocean medium (0.1% yeast extract, 0.05% ammonium chloride, 5 mM Tris pH 7.4 with varying Instant Ocean^®^ Sea Salt (Aquarium Systems Inc, Mentor, OH, USA) concentrations) or LBS [106] medium with varying NaCl concentrations, at 30 °C with aeration. The growth was continued until OD_600_ reached 0.5 (midexponential phase), and then 25 mL of bacterial culture was sampled, centrifuged and resuspended in a lysis buffer (1.5% SDS, 0.1 M DTT, 0.1 M Tris-HCl pH 7.6) in a volume corresponding to 10 x bacterial pellet. The samples were incubated for 5 min at 95 °C, and then kept at −20 °C until further analysis. The doubling per hour for each salinity condition was calculated as follows: doubling/hour = 60/(LOG(2)*((t2) − (t1))/LOG(OD2/OD1)), where t1 and t2 are the time points between which the growth is exponential, and OD1 and OD2 are the optical density measurements in these time points.

### 3.2. Protein Digestion

Proteins were digested with the Filter-Aided Sample Preparation (FASP) procedure [107]. Sample protein concentration was assessed spectrophotometrically, and 100 μg of protein was placed on separate Microcon^®^ filters with a 10 kDa cutoff membrane (Merck-Millipore, Darmstadt, Germany). Filters were washed twice with a urea buffer (8 M urea/100 mM Tris-HCl, pH 8.5) by 20 min centrifugation at 10,000× *g* before alkylation of the cysteine residues by incubation with 55 mM iodoacetamide/urea buffer in the dark, for 20 min. Afterwards, the filters were washed multiple times with the urea buffer and a digestion buffer (50 mM Tris-HCl, pH 8.5). Filters were then placed in new tubes filled with a trypsin solution (1:50 enzyme-to-substrate weight ratio) and incubated at 37 °C overnight. Resulting proteolytic peptides were eluted in additional digestion buffer washes and further cleaned up for mass spectrometry analysis with the Stage Tips procedure [108] on in-house-prepared C18 resin tips. In the last clean-up step, peptides were eluted with 100 μL of 1% acetic acid/60% acetonitrile/water. Before mass spectrometry analysis, the samples were condensed to 40 μL by using SpeedVac.

### 3.3. Mass Spectrometry Measurements

Liquid chromatography coupled with tandem mass spectrometry (LC-MS/MS) analysis was conducted in positive ion mode on the Triple TOF 5600+ instrument (SCIEX, Framingham, MA, USA) coupled with Ekspert MicroLC 200 Plus System (Eksigent, Dublin, CA, USA). In each measurement, 5 μL of injected peptides was separated on ChromXP C18CL column (3 μm, 120 Å, 150 × 0.3 mm, Eksigent) in a 60 min gradient (11–42.5% buffer B; buffer A: 0.1% formic acid/water, buffer B: 0.1% formic acid/acetonitrile). All samples were registered once in data-dependent acquisition (DDA) analysis, which consisted of a TOF scan in the *m*/*z* range of 400–1200 Da for 100 ms and a product ion scan in the *m*/*z* range of 100–1800 *m*/*z* for 50 ms, resulting in a cycle time of 1.15 s. Quantitative analysis was conducted using high-sensitivity-mode SWATH-MS (Sequential Window Acquisition of all Theoretical Mass Spectra) analysis [109] in 25 overlapping variable width acquisition windows, constructed using SWATHTuner software [110] focused on equalized ion frequency. SWATH-MS analysis consisted of a survey ion TOF scan in the *m*/*z* range of 400–1200 for 50 ms, and following product ion scans in the *m*/*z* range of 100–1800 for 40 ms, resulting in a cycle time of 1.1 s. The mass spectrometry proteomics data have been deposited to the ProteomeXchange Consortium [111] via the PRIDE [112] partner repository with the dataset identifier PXD029628.

### 3.4. Data Analysis

SWATH-MS data analysis was conducted in a way similar to that described before [113]. Files resulting from DDA measurements were searched against protein sets from the UniProt database [114] (proteome IDs: UP000076650 for *S. baltica* M1 strain, UP000000537 for *A. fischeri* ES114 strain and four proteomes—UP000008367, UP000067422, UP000032052, and UP000248176—available for *V. harveyi*; versions from 07.04.2021) with ProteinPilot 5.0.2 software (SCIEX) to construct a spectral library. The resulting group file was imported into PeakView 2.2 (SCIEX) along with SWATH-MS measurement files for joint data analysis. The obtained protein intensity values were normalized by a total area sums (TAS) approach. Statistical analysis was performed with Perseus 1.6.15 [115]. Intensity values were log_2_-transformed. Principal component analysis (PCA), hierarchical clustering, and Pearson correlation of samples were performed, and their results were used to exclude potential outliers. At least two biological and technical replications were used in *t*-tests. Differences in protein concentrations with permutation-based FDR (*q*-values) under 0.05 were considered statistically significant. Proteomic data were analyzed with Perseus software [115]. Data were visualized using web-based tools: PlotsOfData Shiny app [116], VolcaNoseR [117] and InteractiVenn [118]. Gene ontology analysis was performed with GSAn 1.0.5 tool [119] using UniprotKB IDs generated with Retrieve/ID mapping tool from UniProt [120] as an input. Gene Ontology (GO) terms and annotations (.gaf files) for bacterial species—*Shewanella baltica* (NCBI:txid62322), *Vibrio harveyi* (NCBI:txid669), and *Aliivibrio fischeri* (NCBI:txid668)—were obtained using the QuickGO web-based browser [121] (accessed on 22 August 2021).

## 4. Conclusions

Analysis of three bacterial strains, representatives of various marine habitats, revealed their response at the proteomic level to alterations in salinity conditions. We found that bacteria adjust at all levels of their biological processes, from DNA topology through gene expression regulation and proteasome assembly, to transport and cellular metabolism. On the one hand, it is surprising that seemingly similar adaptation strategies are observed for the hypo- and hyper-osmotic conditions (as is evidenced by clustering presented in Figure 1C). On the other hand, it is not surprising, since growth rates at these salinities are similar, and we are viewing the proteome when the bacteria are not stressed, but fully adapted to the new conditions. Interestingly, the least number of changes was observed for *S. baltica*, indicating either the least adaptability potential, or, quite contrary, a smooth transition to living under unfavorable conditions. We are aware that the weakness of this study is the limiting of the protein analysis to the bacterial pellet itself, while secreted proteins could be lost. This would explain why some proteins are missing in the analysis. The data could also be extended to validation by RT qPCR or Western blot, and in further studies we plan to extend our analysis with these methods. Our results show that salinity challenge affected translation, indicating that downregulation of translation could increase the overall accuracy of protein synthesis and protein folding, thus improving protein homeostasis and stress adaptation. However, enzymes involved in protein synthesis can have more than one function in the cell, and regulation of their expression could be important for bacterial development in a changing environment or acquisition of a new niche. We found that adaptation to salinity challenge in bacteria clearly involves accumulation of DNA-binding proteins and possibly increased polyamine uptake, and we hypothesize that they are used to coat and protect the nucleoid to counteract adverse changes in DNA topology due to ionic shifts. We suggest that this may be the major mode of action to maintain DNA structure and function in the cell under suboptimal salinity conditions.

## Data Availability

Data are available via ProteomeXchange with identifier PXD029628.

## Supplementary Materials

The following are available online at https://www.mdpi.com/article/10.3390/ijms23020619/s1.
