# Supplementary material for: Three Microbial Musketeers of the Seas: Shewanella baltica, Aliivibrio fischeri and Vibrio harveyi, and Their Adaptation to Different Salinity Probed by a Proteomic Approach
Source: Int J Mol Sci. 2022 Jan 6;23(2):619. doi: 10.3390/ijms23020619 (PMC8775919; doi:10.3390/ijms23020619)
Supplement: Supplementary file 1 [file ijms-23-00619-s001.zip › Supplementary Figures Kloska et al.pdf]

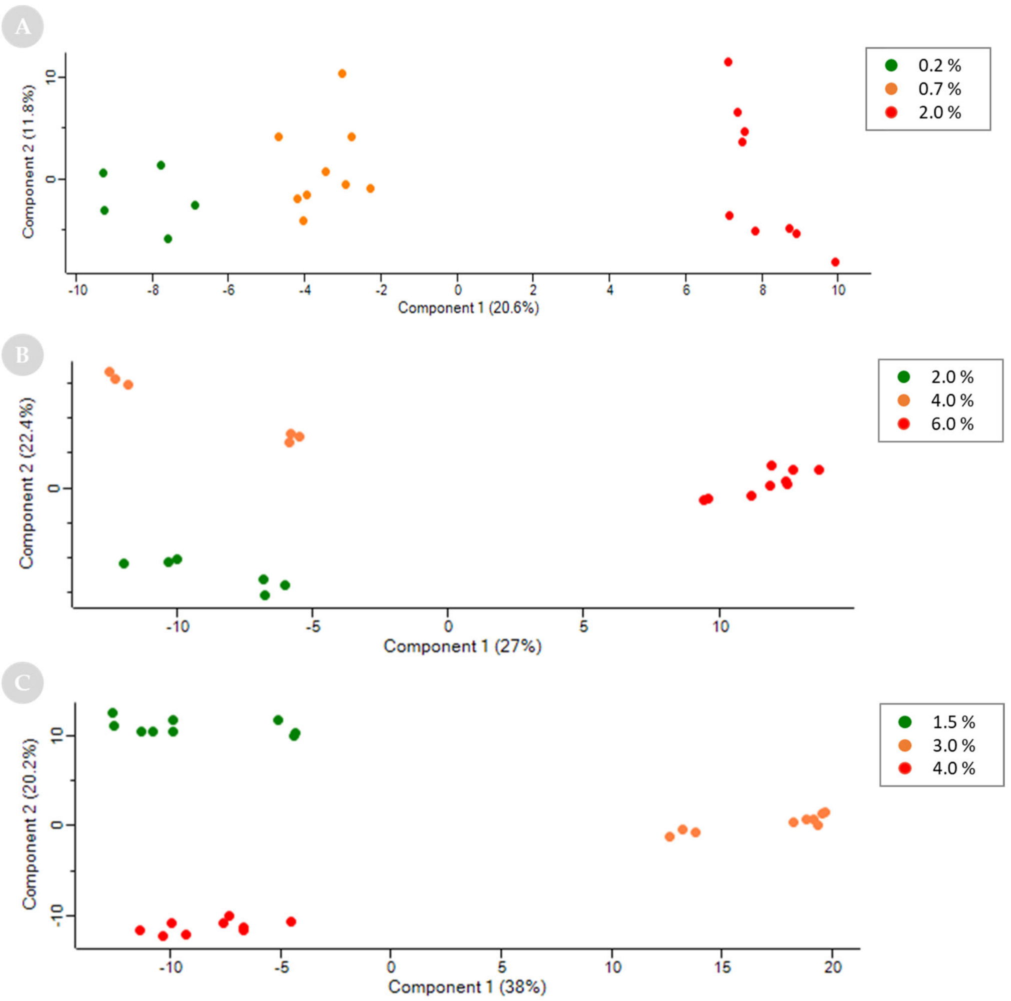

Supplementary Figure S1. Principal component analysis (PCA) of proteomic data. Biological and technical replicates obtained for (A) *S. baltica*, (B) *A. fischeri* and (C) *V. harveyi* grown at low (green dots), optimal (orange dots) and high (red dots) salinity are presented.

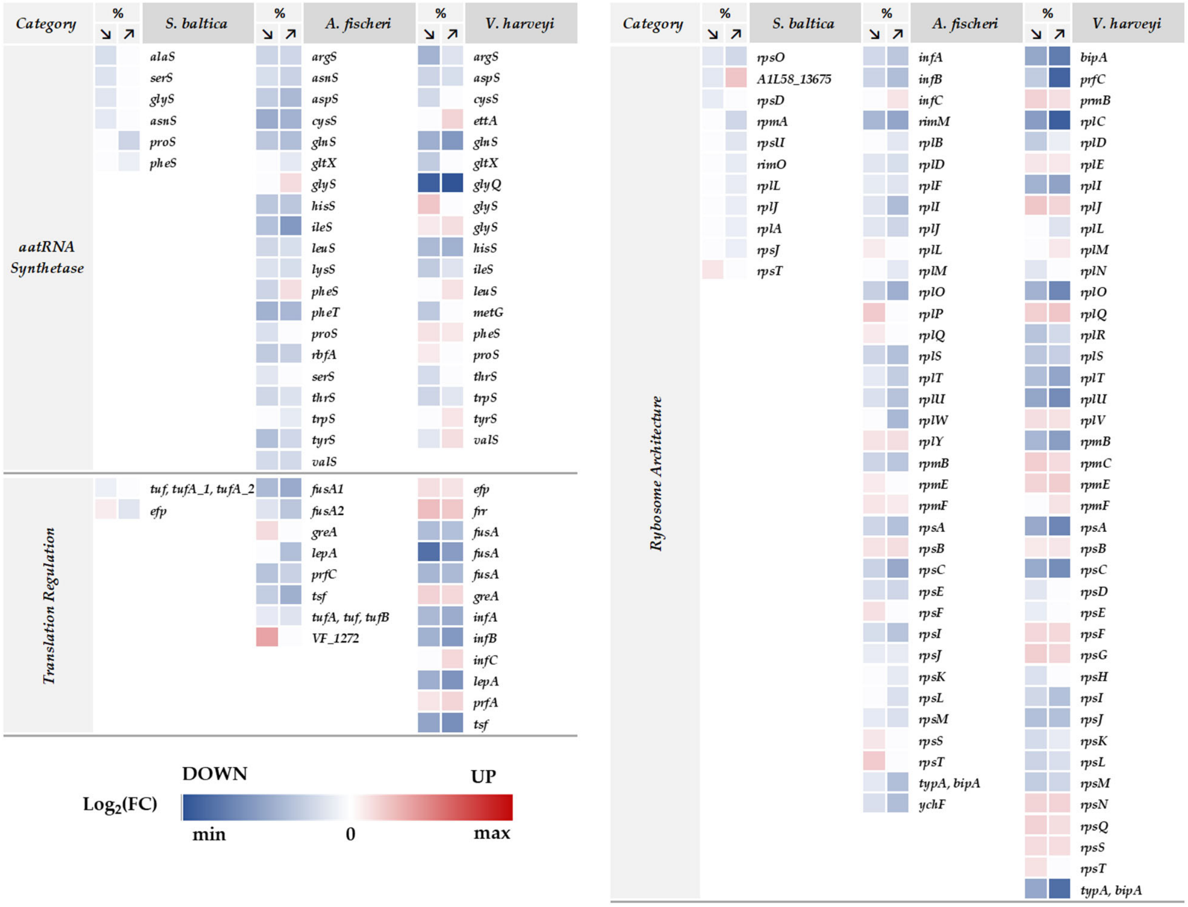

Supplementary Figure S2. Heatmaps of abundance of selected translation-related proteins in *S. baltica*, *A. fischeri*, and *V. harveyi* following low and high salinity stress. In the header, up and down arrows indicate low and high salinity conditions, respectively. The hue of blue or red colors indicates the expression level (log_2-transformed fold change (FC) values) of selected proteins relative to optimal salinity; the corresponding gene names are used to designate proteins. Exact protein fold change values can be found in the Supplementary Table S2.
